# Perspectives of healthcare providers on telemedicine implementation during the COVID-19 pandemic in Colombia: a mixed-method study

**DOI:** 10.3389/fpubh.2025.1643085

**Published:** 2025-08-01

**Authors:** Susana Orrego Villegas, Eric Adjei Boakye, Jafet Arrieta, Karine Posada España, Melisa Naranjo Vanegas, Gloria Pardo Pino

**Affiliations:** ^1^Harvard Medical School, Boston, MA, United States; ^2^Universidad CES, Medellín, Colombia; ^3^Seguros Sura Colombia, Medellín, Colombia; ^4^Department of Public Health Sciences, Henry Ford Health, Detroit, MI, United States; ^5^Institute for Healthcare Improvement, Boston, MA, United States; ^6^Harvard TH Chan School of Public Health, Boston, MA, United States; ^7^Bioscience Center Sura, Medellín, Colombia; ^8^Universidad Tecnológica del Chocó Diego Luis Córdoba UTCH, Chocó, Colombia; ^9^Organización Panamericana de la Salud (PAHO), Washington, DC, United States

**Keywords:** telemedicine, COVID-19 pandemic, Colombia, healthcare provider, mixed-methods, digital health, health equity, health systems

## Abstract

**Background:**

The COVID-19 pandemic accelerated the adoption of telemedicine (TM) in Colombia as a tool for delivering healthcare for both chronic and non-chronic conditions. This study aimed to explore the perspectives of healthcare providers, directors, and managers on TM implementation within a major health plan provider covering 15% of the Colombia population.

**Methods:**

Mixed-methods study was conducted, combining surveys and in-depth interviews with medical professionals and decision-makers. Thematic analysis of qualitative data and statistical analysis of survey responses provided insights into the challenges and opportunities of TM during the pandemic.

**Results:**

Healthcare providers recognized TM as a valuable tool but identified key challenges to its optimization. Three main strategies were proposed: (1) enhancing medical training to improve remote diagnosis, (2) integrating wearables and digital technologies to support clinical decision-making, and (3) fostering a culture of TM use among patients and providers to ensure acceptance and sustainability.

**Conclusion:**

Telemedicine facilitated equitable healthcare delivery and contributed to reducing the digital gap in Colombia. However, to ensure long-term viability, national policies must improve internet connectivity and strengthen primary care infrastructure, particularly in underserved regions.

## Introduction

The integration of digital technologies into healthcare has been a long-standing objective of global health organizations. In 1997, the World Health Organization (WHO) recognized the potential of tematics to transform healthcare delivery, emphasizing the need for technology-driven strategies to achieve equitable health access worldwide (1). Over the past three decades, researchers and policymakers have worked to incorporate telemedicine (TM) into clinical practice, aiming to improve medical decision-making and expand healthcare access. However, challenges such as technological infrastructure, regulatory frameworks, and the digital divide have limited its widespread adoption (2–4).

Before the COVID-19 pandemic, Latin America displayed substantial disparities in telemedicine uptake (5, 6). Countries like Chile and Uruguay adopted telehealth strategies early and reached implementation rates over 60% in hospitals, while adoption in countries such as Argentina, Mexico, and Peru ranged between 30 and 50% (7). In contrast, Colombia’s adoption of telemedicine was estimated at only 25–26% of healthcare institutions, despite having a supportive regulatory framework, due to barriers such as insufficient broadband access, provider resistance and limited reimbursement policies (6, 7).

In Colombia, the implementation of TM remained limited before the COVID-19 pandemic. Between 2007 and 2019, the Ministry of Health and Social Protection (MHSP) introduced regulations to promote TM services, allowing a few public and private health insurance providers to offer remote medical consultations (8–11). Among these was Sura, one of the largest insurers in the country, covering approximately 13.3 million people and serving 5.9 million patients under its public coverage (12). Despite these efforts, TM was not a primary mode of healthcare delivery, and its use was sporadic and restricted to specific clinical scenarios.

The outbreak of COVID-19 in early 2020 created an urgent need to restructure healthcare delivery. The Colombian government implemented a nationwide quarantine from March 24 to July 15,2020, significantly disrupting routine healthcare services (7). Elective surgeries and non-emergency consultations were suspended, increasing the risk of care delays and worsening health outcomes for patients with chronic conditions (13). In response, TM rapidly emerged as a critical tool for maintaining healthcare access. The government incentivized the expansion of TM services, leading to over 3 million virtual consultations within the first 2 months of the pandemic and more than 42 million by January 2021 (13, 14).

Despite this unprecedented adoption, key questions remain regarding the effectiveness, accessibility, and long-term sustainability of TM in Colombia. Challenges such as disparities in digital literacy, uneven access to internet connectivity, and the need for provider training in remote diagnostic continue to shape the future of TM in the country (7). Moreover, while TM has proven valuable during the pandemic, its role in post-pandemic healthcare models remains uncertain.

To address these gaps, this study aims to assess the perspectives of healthcare providers and administrators on the impact of telemedicine during the COVID-19 pandemic. By identifying the benefits, limitations, and areas for improvement, this research seeks to inform policies that ensure the sustainable and equitable integration of telemedicine into Colombia’s healthcare system.

## Methods

### Study design and setting

This study employed a mixed-method approach, emphasizing the qualitative component with the objective of characterize the experiences and perceptions of health plan leadership and medical teams who managed care during the COVID-19 pandemic at a leading healthcare insurer in Colombia. The study aimed to generate in-depth, contextualized understanding of organizational and clinical adaptations during the pandemic.

SURA Colombia is a private health insurer offering contributory public health insurance (EPS SURA), occupational risk insurance (ARL SURA), and private supplementary health policies (Polizas SURA). SURA operates nationally, with Primary Care Units (PCUs) primarily concentrated in urban centers within central and northern Colombia.

Colombia’s health system is underpinned by Universal Healthcare Coverage (UHC) since 1994, mandating enrollment in EPS plans. Employment status determines ARL affiliation, and individuals may additionally purchase private insurance. Each patient is assigned to an EPS and corresponding PCU (referred to locally as IPS).

### Participants and recruitment

Purposive sampling strategy was implemented to select five managers and two medical directors, all of whom held significant decision-making responsibilities during the COVID-19 response at SURA.

Additionally, seven physicians who transitioned from in-person care to telemedicine during the pandemic were recruited through targeted email invitations facilitated by SURA’s Human Resources and Research teams. Eligible participants were full-time employees actively involved in COVID-19 care delivery. Informed consent was obtained electronically prior to participation. Recruitment and data collection were conducted between August and October 2022.

### Data collection

Data collection was conducted remotely between August and October 2022 using Harvard Secure Zoom, a videoconferencing platform that ensured confidentiality and data protection. The principal investigator (PI), a bilingual medical doctor and master’s candidate at Harvard Medical School, personally conducted all interviews and moderated the focus group to maintain consistency and minimize potential interviewer bias.

The qualitative dataset comprised five individual semi-structured interviews with managers and directors, each lasting 60 to 75 min. These interviews explored strategic decision-making processes, challenges faced in managing health plan operations, and organizational adaptations to the pandemic context. The semi-structured interview format allowed the PI to probe for detailed responses and emerging themes, facilitating a deep understanding of participants roles’ and experiences.

In addition, a focus group discussion was held with seven physicians who had shifted from traditional in-person care to telemedicine modalities during the pandemic. The group interaction fostered dynamic dialog about clinical workflows, technology integration, and patient engagement challenges in the telehealth setting. To ensure participant anonymity and promote open communication, cameras were kept off and participants were anonymized through assigned numbers. The sessions lasted approximately 90 min.

A hybrid session was also conducted with two medical directors and one manager, combining features of individual interviews and focus group discussions. This format enabled in-depth exploration of operational and clinical leadership challenges while allowing participants to interact and expand upon each other’s perspectives.

All interviews and group discussions were audio-recorded with participant consent, transcribed verbatim in Spanish by the PI, and subsequently translated into English by the same investigator to ensure conceptual fidelity. This uniform approach to data collection, transcription, and translation contributed to methodological rigor and data integrity,

### Data analysis

The PI developed a detailed codebook through an iterative process involving testing, revision, and refinement to guide systematic coding of the qualitative dataset using Dedoose software. Coding quality was maintained through continuous oversight and team discussions.

An inductive thematic analysis was conducted to identify patterns and categories reflecting participants ‘experiences and perceptions regarding telemedicine implementation and pandemic-related care adaptations. The PI created comprehensive category descriptions and supported findings with illustrative quotes.

The second author independently reviewed the preliminary themes, facilitating iterative refinement and deepening of analytic categories through revisiting the transcripts. This collaborative approach enhanced the credibility and trustworthiness of the results.

## Results

We conducted one focus group discussion with seven medical doctors who provided telemedicine (TM) care between March 2020 and June 2021. Part of the medical team continued delivering care via TM during the interviews in August 2022. Additionally, we performed individual interviews with five managers. One manager and two medical directors participated in both the focus groups and individual interviews.

From the data, we identified nine major themes describing: the medical team’s experience using TM, patient factors linked to greater engagement and success with TM, and challenges faced by health plan leadership in implementing TM.

### The role of physical contact in care provision

The abrupt transition from in-person consultations to telemedicine during the COVID-19 pandemic caused anxiety among clinicians, who feared diagnostic errors due to the lack of physical examination. Participants emphasized a deeply ingrained culture of face-to-face patient contact. Both providers and patients expressed concerns that the absence of physical touch compromised care quality.

*“…I felt like I couldn't do anything because I was afraid that I wouldn't take good care of the patient, or I would make a mistake. What if it was not that diagnosis? That scared the hell out of me.” Medical Doctor #6*

*“…The big challenge is to change culture. We come from a traditional medicine where the physical examination and where the patient always wants to meet someone who touches him… So, it is hard for the patient to understand that there are things that can actually still be done that don't need such a thorough physical examination.” Medical Doctor #3*

Over time, supported by guidance from managers and supervisors, some clinicians adapted resourcefully by incorporating alternative diagnostic tools such as patient-submitted photos or videos, and refining their communication techniques.

*“Over time I realized that I have an enormous capacity for adaptation, and I believe that all human beings do. You adapt and learn to work with what you have. You start inventing ways to look at things you can't see. For example: I asked the patient to send me a photo, asked for better descriptions or sent a photo and told them if it looked like what they were presenting. You start looking for ways to make things easier and adapting to it.” Medical Doctor #6*

Participants emphasized that a cultural shift is needed among providers and patients to move beyond the notion that quality care requires physical presence, recognizing that telemedicine can deliver effective, satisfactory care.

*“I believe that the challenge in telemedicine today for me is to try to provide care that the patient prefers. How can we become the preferred channel for patients? During the pandemic, when the health emergency was at its peak, patients had to resort to telemedicine because there were no other options. However, the challenge today is how we can be responsive from telemedicine, how Telemedicine can provide quality and relevant care to patients in a way that makes them feel satisfied. I think that's the daily challenge, where the patient already has the option to choose between in-person or virtual consultations and decides to choose virtual care.” Medical Doctor #4*

### Telemedicine and increased medical expenditure

To compensate for the absence of physical examination, physicians reported ordering more laboratory and diagnostic tests, which managers identified as driving increased healthcare costs.

*“I have also ordered exams, to complement the lack of the physical exam. I have ordered X-rays and ultrasounds.” Medical Doctor #7*

*“There are situations where I feel that requesting additional exams is necessary, especially when dealing with conditions such as an injury, knee pain, or a mass. These types of cases require a physical examination to determine the appropriate course of action. Without this, it is better to send additional diagnostic tests to confirm the diagnosis before referring the patient to a specialist.” Medical Doctor #6*

*"Today, the cost of virtual healthcare is almost 3.5 times higher than in person visits. It's not just the cost of the consultation itself, but everything that is being derived from it. The physician, in an effort to resolve the patient's healthcare needs, tends to increase referrals to specialists, diagnostic exam…because we lack the tools to provide a more effective resolution”. Manager 2*

### Protecting the wellbeing of the medical team

Participants acknowledged the psychological and physical strain on medical staff during the COVID-19 pandemic. Managers implemented risk assessments to identify staff with pre-existing conditions, assigning them to telemedicine roles to reduce exposure risk.

*“… We evaluated the risk of the health care workers and found they had high vulnerability. A diabetic, a hypertensive, someone over 50 years old, we told them not to come to work in person. We put those doctors to work in telemedicine, and it was tough for them. Many said, 'They won't let me go because I'm old or because I have a risk factor, because I'm overweight.' And it was difficult for many to accept that we marked them as someone more susceptible to having a higher risk of morbidity and mortality from COVID. Some liked it, and others didn't.” Manager 3*

Managers also prioritized emotional support and workload management by creating spaces for breaks, rest, and therapy to safeguard staff wellbeing.

*“…It was very challenging because not only were we under pressure and uncertainty, which was a constant for us, but also because we had to be like emotional caretakers for the team… So, we had to provide therapy and talk a lot with the team. In the beginning, for example, we started with a level of work that didn't stop, we didn't stop, we didn't stop. I remember that during Holy Week (April 2020), going over that process from start to finish, we met twice a day. Well, we did not stop. And then we reached a point where we said: this is not going to be a 100-meter race, it's a marathon. So, since it's a marathon, we need to regulate ourselves and regulate the team, rest, take days off, and make sure people could rest.” Manager 5*

### Urban versus rural telemedicine delivery

Participants noted that while quality of medical diagnosis was consistent across urban and rural patients, rural patients faced challenges such as limited internet connectivity, often restricting consultations to phone calls rather than video.

*“If we are talking about a rural person and a person from the city, then they're equal to me. But if we're talking about technology, communication, or connectivity, it's different because sometimes in rural areas there may not be as much access to connectivity or signal.” Medical Doctor # 5*

*“There is limited access to the internet in many rural areas. This makes it difficult to access telemedicine” Manager 2*

Administrators highlighted telemedicine’s value in providing access to specialists for patients located nationally and internationally but noted logistical challenges in coordinating follow-up care and delivering supplies in resource-limited rural settings.

*“We managed to make it cross borders, in other words, not only were patients seen all over Colombia and abroad through the use of telemedicine, regardless of where they were located. They only needed to have access to the internet or telephone line.” Medical Director # 1*

*“…I would say that access from rural areas was not a problem for us; it was more of a challenge to transport supplies (such as oximeters and personal protective equipment for healthcare workers) for those patients who were considered at risk. This was a much more complex logistical issue, rather than an issue of access to telemedicine.” Manager 3*

Managers emphasized telemedicine’s potential to scale healthcare access equitably across Colombia, improving mortality and health outcomes in underserved regions.

*“The operating model makes us much more scalable and allows us to reach many more territories in Colombia than we are currently present in, and in that way, we can generate a much greater impact on Colombian society and generate results for the entire population and be much more equitable… If we had been present in more regions during the pandemic and implemented our model, we would have been able to have a much greater impact on mortality and health outcomes.” Manager 2*

To improve access, alternative telemedicine channels such as toll-free numbers, apps, and WhatsApp were implemented, accommodating patient preferences and technological limitations.

*“… We had a perfect model where everyone would just connect easily to a video call. However, we encountered technological problems that were normal, and we lost that channel. We had to open other options to access to telemedicine, such as the customer service line and WhatsApp. So, we ended up doing telemedicine, but we had to open many channels to provide medical attention to the patients according to their preferences.” Manager 4*

### Impact on clinician work-life balance

Clinicians reported personal benefits from providing care via telemedicine, including improved work-life balance, financial savings, and enhanced wellbeing.

They highlighted reduced comminating costs and work-related expenses, increased schedule flexibility, opportunities for exercise and family time, and decreased stress and anxiety.

*“It has been very beneficial for me. I lost weight, eat better, and have more time for myself and exercise, were things I could not do before due to mobility, transportation, and time constraints. I started to see the positive side of telemedicine because I had more time for myself. Before the pandemic, I was very sick with gastritis and high triglycerides, but all of that improved because I was able to take better care of myself, had more time for myself, and it was a radical change for me.” Medical Doctor #6*

*“I feel that I am improving my workplace a lot, because I can adapt it in my house to my needs. Here I have the right chair for me, and I had my desk made right for me and I feel like I have my space. When I worked at headquarters, there was no space of my own, and I had to share different offices with several colleagues. I was too stressed when I had to go to the office and my partner was late (because it causes stress for me, if I have my appointment at 14:00, it started at 14:10 or 14:20).” Medical Doctor #1*

### Responsiveness to emerging trends and pandemic preparedness

Participants emphasized that pre-pandemic telemedicine implementation was critical to maintaining healthcare delivery during COVID-19, preventing healthcare system collapse.

*“Can you imagine if we hadn't had telemedicine? All those patients going out on the street to seek medical attention?... I don't want to imagine that scenario. Well, the chaos would have been worse, especially because there was a lot of anxiety on the part of the patients, who only with telemedicine care, would calm down and should not go to headquarters or clinics to seek care, at the risk of infecting others or infecting themselves… Having the possibility to use telemedicine to attend to 95% of patients, validate the risk of complications they had, and leave health care facilities for those who really needed them, helped us to be very organized… When COVID arrived, if it hadn't been for telemedicine, rather, all the health infrastructure that this country had would have collapsed, especially SURA's.” Manager 5*

Clinicians described working amid rapidly evolving international guidelines and high uncertainty, requiring flexibility and rapid adaptation.

*“Due to the nature of the business, there's always been some degree of uncertainty that we're accustomed to identifying and managing as a trend. However, the level of uncertainty changed and completely shifted. And it wasn't the kind of uncertainty that we were used to identifying and managing, but it started changing in a matter of hours. We had a problem today and believed we solved it, but it was no longer the problem of tomorrow. So, that level of uncertainty and the constant change within a short period of time required us to mobilize and be able to do everything.” Medical Director # 2*

Participants acknowledged gaps in preparedness and expressed a need for greater anticipation, increased staffing, and upgraded infrastructure based on early international outbreak data.

*“Well, first of all, we needed to have much more capacity for anticipation. If we hadn't been caught off guard, I think that monitoring what was happening in Italy, Spain, England, and China wasn't enough. So, we could have been more proactive. Secondly, I believe that we could have handled processes differently. Having enough doctors or hiring telemedicine and call center services from expert companies and third parties is much easier.” Manager 3*

### The future of telemedicine as an “expert ally”

Participants shared that as the pandemic waned, and in-person visits became more available, they noticed that telemedicine usage began to drop.

*“… People have been asking for more in-person care than virtual care because they perceive it as being more effective. We set a goal to have between 30% to 40% of virtual care this year, and we achieved it in the first few months of 2022. However, the numbers have been decreasing… The challenge is to transform the culture, but we also need to measure the results and ensure people see that there is no difference between virtual and in-person care for certain issues. We need to understand who and when it's appropriate to provide virtual care." Manager 2*

Having seen the potential of telemedicine for chronic conditions, participants expressed hope that it could be used to support in-home care and bolster preventive medicine. Using telemedicine in this targeted way could help make more efficient use of health resources and relieve congested hospitals and clinics.

*“…Through telemedicine we can enable and provide the peace of mind and security of receiving care at home, which is currently a trend and has been for a long time as a way to decongest clinics and hospitals and to address certain pathologies that do not require hospital-level care. In our country, given the capacity of the healthcare system, this is a fundamental topic.” Medical Director # 1*

To bolster the use of telemedicine as an “expert ally” alongside in-person care, participants explained that telemedicine needed to more closely approximate a face-to-face visit. They believed that this could be achieved by incorporating new technologies, such as wearable devices or cameras, which can monitor patients ‘disease progression or assist in diagnosis. Many participants also envisioned making use of artificial intelligence as a way to connect the daily experience of the patient with the chain of care.

*“I think it will play a role in becoming the expert ally… Telemedicine will enable to have an scenario where the patient trust, that the healthcare system will take care of them.” Manager 5*

*“I believe that in the metaverse, we will have access to tools that enable us to be present virtually, using augmented reality that blurs the line between physical presence and virtual presence.” Manager 2*

*“Telemedicine should be accompanied by wearable devices such as smartwatches and nano cameras. This transformation toward prevention will allow for more efficient use of resources, with the ability to monitor patients remotely. This way, we can work toward the well-being of people, even at a distance, by using telemedicine to connect patients with the necessary health resources.” Manager 1*

To complement the thematic findings, we generated a word cloud based on recurring concepts in participants’ reflections on the evolving role of telemedicine. Words such as *trust*, *efficiency*, *home care*, and *wearables* emerged prominently, highlighting the vision of telemedicine not as a substitute, but as an integrated and intelligent extension of the health system. Participants imagined a future where virtual care is enhanced by emerging technologies—such as artificial intelligence and augmented reality—to monitor chronic conditions, support preventive care, and provide patients with confidence and continuity. The prominence of the term *expert ally* reflects the aspiration for telemedicine to become a trusted partner in a hybrid model of care.

In summary, participants emphasized that the future of telemedicine lies not in replacing in-person care, but in becoming its strategic complement – an expert ally that integrates cutting-edge technologies, supports preventive care and reinforces the continuity of care. For this vision to be realized, efforts must be made to shift cultural perceptions, build patient trust, and demonstrate the tangible value of virtual care in enhancing both access and quality.

## Discussion

This study offers new insights into the perceived advantages and challenges of telemedicine implementation during the COVID-19 pandemic, as experienced by physicians, medical directors, and healthcare managers in Colombia (15–18). Drawing on qualitative data, it outlines a potential roadmap for transforming telemedicine from a temporary solution into a long-term, expert ally in hybrid models of care.

While the health plan did not extend uniformly to all regions, telemedicine services were deployed nationwide as an attempt to bridge structural inequalities, including the digital divide (16, 19). Notably, this is the first study in Colombia exploring the role of telemedicine during the pandemic that is not limited to a single institution or disease domain, thus providing a broader health system perspective.

Participants recognized that telemedicine had enabled continuity of care for a wide range of conditions during a time of extreme health system strain (20). However, as in-person visits resumed post-pandemic, telemedicine usage declined, reflecting both a cultural preference for face-to-face interactions and concerns about diagnostic limitations without physical examination (17, 18). These findings suggest that while telemedicine proved feasible and effective during the crisis, its long-term integration will require intentional efforts across multiple dimensions.

Three key areas of transformation were identified to sustain telemedicine as a valuable component of care delivery: First, clinical adaptation and training are essential; health professionals must be equipped with new competencies to make accurate clinical decisions in virtual settings (21, 22). This includes developing enhanced communication skills and diagnostic reasoning without the benefit of physical examination. Investing in these capabilities is critical to ensure clinical accuracy and patient safety in telemedicine encounters. Second, technology integration plays a pivotal role; the incorporation of wearable devices, remote monitoring tools, and artificial intelligence can support clinical decision-making, facilitate early detection of disease progression, and enhance patient engagement. These tools bridge the gap between virtual and in-person care, enabling clinicians to deliver more personalized and proactive care remotely (23, 24). And finally, a cultural shift and greater patient trust are needed; participants stressed the importance of educating patients on the value and safety of virtual care, demonstrating comparable health outcomes, and positioning telemedicine not as a backup but as a legitimate and often preferred mode of healthcare. System-wide communication efforts and leadership buy-in will be key to establishing telemedicine as a normalized part of the healthcare experience (24–26).

Additionally, we recognize the value of interpreting these findings through established theoretical lenses. Applying Lehoux et al. theory of use of telemedicine, we can appreciate that successful integration requires compatibility with clinicians’ routines and practices (27). Furthermore, drawing on Rowther et al. and their socio-technical perspective on telemedicine use, we highlight the importance of trust, relationship, and the co-creation of care processes between patients and providers. These frameworks enrich our understanding of the conditions that facilitate sustained adoption and scaling of telemedicine solutions in real-world healthcare systems (28).

Participants also envisioned a future in which telemedicine is integrated into immersive digital ecosystems—including augmented reality and metaverse-based environments. Such innovations could help humanize virtual care, allowing clinicians and patients to interact in more engaging and responsive ways while preserving the efficiency and flexibility of remote services.

From a policy perspective, participants underscored the need for governmental action to expand internet connectivity in underserved regions and reinforce the capacity of primary healthcare systems. These investments are essential to ensure continuity of care when virtual services must be supplemented by in-person referrals, diagnostics, or interventions. Without robust digital infrastructure and integrated referral systems, telemedicine may inadvertently widen existing healthcare disparities.

## Limitations

While this study offers valuable insights into telemedicine implementation during the COVID-19 pandemic, it has some important limitations. The findings reflect the experiences of healthcare professionals within a large private health insurance provider operating mainly in urban areas, which may not capture the full range of experiences across different care settings and populations. Furthermore, as a qualitative study, its scope and transferability are inherently limited, and it does not assess quantitative clinical or economic outcomes. These constraints highlight the need for further research that tests the generalizability of these findings.

## Conclusion

Telemedicine has the potential to become a central component of Colombia’s healthcare model, but realizing this vision requires structural, technological, and cultural transformation. Strengthening clinical training, investing in smart technologies, cultivating patient trust, and improving digital infrastructure are all essential steps.

Building on this work, future research could expand to include rural and public sector institutions as well as the voices of patients themselves, enriching the diversity of viewpoints and uncovering context-specific strategies for telemedicine adoption. Additionally, incorporating quantitative and mixed methods approaches would allow for broader generalizability and evaluation of clinical and economic outcomes.

With appropriate policy support and sustained innovation, telemedicine can evolve from a crisis-response tool into a long-term “expert ally” for equitable, efficient, and human-centered healthcare.

## Data Availability

The raw data supporting the conclusions of this article will be made available by the authors, without undue reservation.
